# Ten simple rules for switching from face-to-face to remote conference: An opportunity to estimate the reduction in GHG emissions

**DOI:** 10.1371/journal.pcbi.1009321

**Published:** 2021-10-18

**Authors:** Valentin Guignon, Catherine Breton, Jérôme Mariette, François Sabot, Julien Fumey, Vincent Lefort, Anna-Sophie Fiston-Lavier

**Affiliations:** 1 Bioversity International, Montpellier, France; 2 South Green Bioinformatics Platform, Bioversity, CIRAD, INRAE, IRD, Montpellier, France; 3 University of Toulouse, INRAE, UR MIAT, Castanet-Tolosan, France; 4 DIADE, University of Montpellier, CIRAD, IRD, Montpellier, France; 5 Société Française de Bioinformatique Executive Board, Paris, France; 6 LIRMM UMR 5506, CNRS, Université de Montpellier, Montpellier, France; 7 Institut Français de Bioinformatique, CNRS UMS 3601, France; 8 Institut des Sciences de l’Evolution de Montpellier (UMR 5554, CNRS-UM-IRD-EPHE), Université de Montpellier, Montpellier, France; 9 Institut Universitaire de France (IUF), Paris, France; Dassault Systemes BIOVIA, UNITED STATES

## Abstract

In 2020, the world faced the Severe Acute Respiratory Syndrome Coronavirus 2 (SARS-CoV-2) pandemic that drastically altered people’s lives. Since then, many countries have been forced to suspend public gatherings, leading to many conference cancellations, postponements, or reorganizations. Switching from a face-to-face to a remote conference became inevitable and the ultimate solution to sustain scientific exchanges at the national and the international levels. The same year, as a committee, we were in charge of organizing the major French annual conference that covers all computational biology areas: The “Journées Ouvertes en Biologie, Informatique et Mathématiques*”* (JOBIM). Despite the health crisis, we succeeded in changing the conference format from face to face to remote in a very short amount of time. Here, we propose 10 simple rules based on this experience to modify a conference format in an optimized and cost-effective way. In addition to the suggested rules, we decided to emphasize an unexpected benefit of this situation: a significant reduction in greenhouse gas (GHG) emissions related to travel for scientific conference attendance. We believe that even once the SARS-CoV-2 crisis is over, we collectively will have an opportunity to think about the way we approach such scientific events over the longer term.

## Introduction

JOBIM (for “Journées Ouvertes en Biologie, Informatique et Mathématiques*”*) or the “Open Days in Biology, Informatics and Mathematics,” is the major French annual conference that covers all computational biology areas, with a special attention to young bioinformaticians. The July 2020 edition was planned in Montpellier, France since mid-2017 in agreement with the steering committee and the French Society of Bioinformatics (“Société Française de Bioinformatique” (SFBI): (https://www.sfbi.fr/executiveboard)). As for any other scientific congress, we set up an organizing committee, which was composed of nearly 40 scientists from 9 institutes, representing Montpellier’s bioinformatics research landscape, with the local help of the Agropolis International Association (https://www.agropolis.fr/). Expecting more than 400 attendees, our first action in 2018 was to book the most spacious and requested conference center in Montpellier. From January 2019, regular meetings were organized, focusing on fundraising, website management, and scientific aspects. Later on, we split the organizing committee into several task forces in order to organize the social events, caterers, possible places to visit for the gala event, animation bands, goodies, carbon footprint reductions, and other matters typical of on-site conferences. In early 2020, the initial budget plan was set, and a list of new sponsors to prospect was defined.

Due to the Coronavirus Disease 2019 (COVID-19) pandemic situation, we started discussing the possibilities of cancelling, rescheduling, or switching to a remote conference as early as the beginning of March 2020. After the lockdown that started in France on March 17, we seriously considered switching to a remote format with the support of the SFBI board. Many issues were raised such as the ability to cancel the already blocked reservations, in particular the conference center, which represented an important part of our initial budget (approximately 30%; 55 K€). Nevertheless, we had to wait until early April for the official announcement of cancellation of public gatherings to initiate changes to the conference. We conducted several surveys within our community (organizing and scientific committees, invited speakers, SFBI board and members, etc.) to find out whether people were ready to attend a remote edition. The strong support of the community and of the SFBI motivated us to pursue this switch in the organization of JOBIM 2020. The challenge did not only lie in the organization but also in the reduced amount of time we had to do it (less than 3 months) and in finding and creating new solutions that did not exist. In the end, we succeeded and had more attendees than initially planned (680 attendees).

The COVID-19 crisis induced a new “stop-and-go” lifestyle, in which no one is able to predict the near future. All conference organizers thus need to be prepared to switch from a regular face-to-face format to a remote one. Interestingly, the public health crisis also reinforces climate crisis awareness, which is a positive outcome. All this pushes us to think more and more about new ways of organizing conferences in the future, reducing our greenhouse gas (GHG) emissions and reinventing our social interactions.

Two previous papers proposed a set of “10 simple rules” for implementing online conferences from scratch [1,2]. However, the current pandemic crisis shows that we have to be reactive and able to switch from face-to-face to purely online events. Thus, we propose 10 simple rules based on our experience to organize such a switch.

### Rule 1: Handle the core organizational tasks

From the very beginning of the organizing work, it is important to identify the core organizational tasks that have to be done whatever the conference configuration. Thus, you will need to be well organized with dedicated committees and teams (e.g., scientific committee, organization committee, budget, IT, training teams, conference session teams, defined roles, up-to-date document sharing, etc.) and good project management practices. Regular meetings have to be planned to move forward in the organization (involve people, get motivated, measure organization progress, and list remaining tasks).

A key element for a well-organized conference is to have an early setup of groups with clearly assigned tasks. Within the organizing committee, specific tasks have to be assigned to dedicated people. This includes for instance budget management, applying for grants, the choice of IT tools used to communicate and to share documents, including the conference website. Then, a provisional schedule has to be set up with milestones in order to coordinate each task group. These tasks are essential for the conference organization no matter whether it is a face-to-face or a remote event.

### Rule 2: Validate the feasibility—Check if you can do it

Before switching from a face-to-face to a remote format, you need to make sure that the speakers and attendees are still willing to participate. You have to check whether the speakers still agree to give a talk by webcam. Furthermore, it is important to get the speakers agreement for video recording and diffusion. Thanks to videoconference recording capabilities, each talk may be recorded and made available for catch-up sessions.

**Fig 1:**
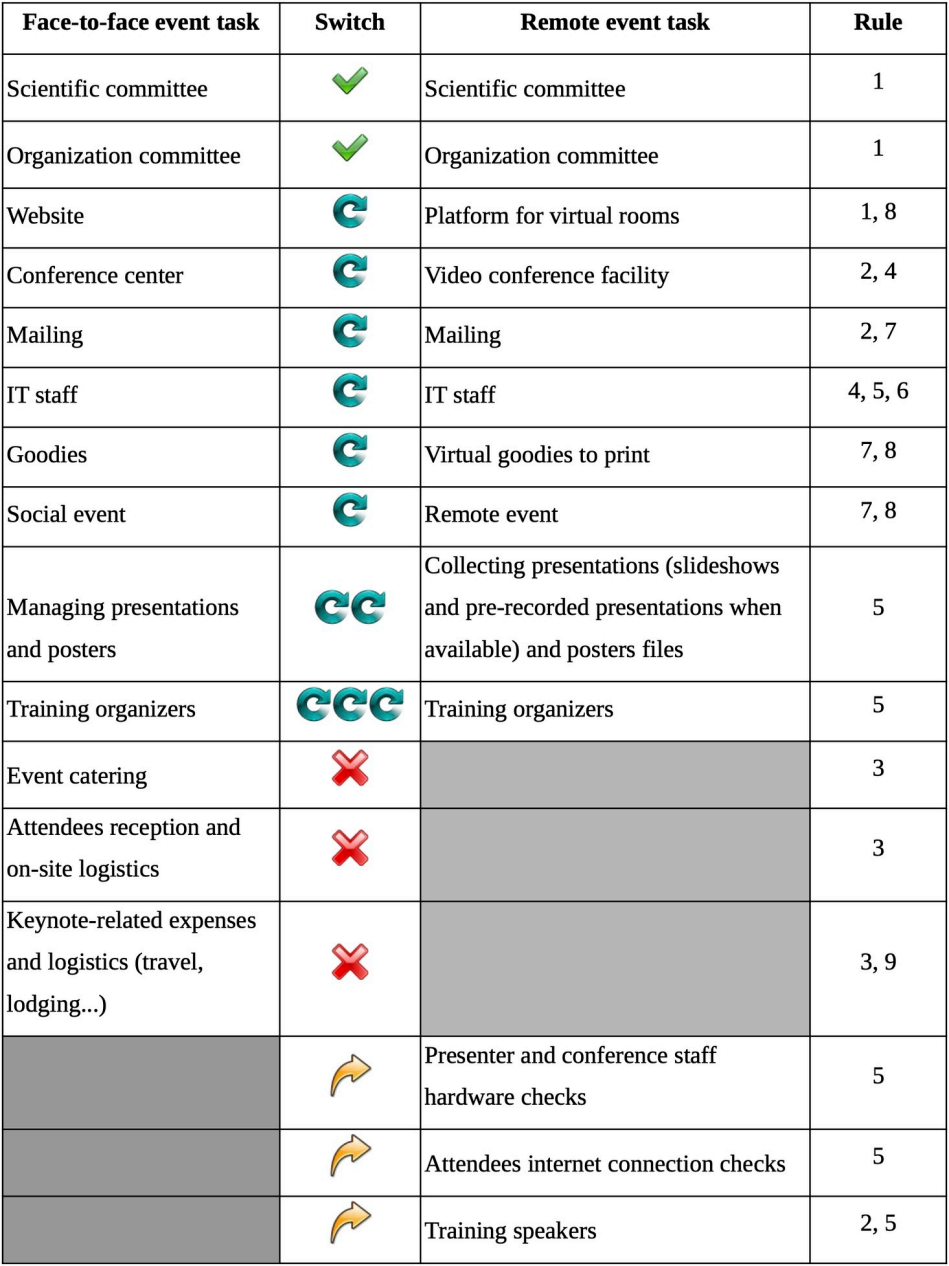
Core tasks mapping. No changes are represented by green check marks. Conserved tasks with changes (more or less) are represented by blue circular arrows. Not conserved tasks are represented by red crosses. Tasks specific to remote are notified by yellow arrows.

Another important point for feasibility is to check that the grants obtained to support the conference are compatible with a remote organization. Indeed, awarded grants may be subject to some conditions. For instance, some grants are dedicated to travel expenses of keynote speakers. By switching to a remote conference, that grant may be lost and consequently lead to a budget reduction. You also need to make sure that the members of the scientific and organization committees still want to be part of the organization. Even if core tasks are maintained, some tasks might change or be newly created (see Fig 1). Be aware that this new organization might naturally exclude some members of the committee and require additional needs. Even if the total cost is definitely affected by the new organization, mainly due to the absence of social events and food expenses, the technical services required might be expensive. Also, as the switch was an unseen situation, some sponsors do not think about the new expenses due to the new technical services and may cancel their support. By consequence, we may end up with less funding and then have to ask for registration fees. For JOBIM 2020, as none of the sponsors cancelled their financial support, the registration was free for the members and only 50 € for the nonmembers of the SFBI.

### Rule 3: Redefine and reassign tasks

When switching to a remote conference, some tasks planned for the face-to-face format become irrelevant, while most of them will have to be adapted or transformed, and others will arise. While the scientific or organization committees might not necessarily change, several tasks need to be more or less adapted. The most important change concerns the training of the organizers and speakers for the handling of the new technical solutions. It will also be challenging to collect all the presentations and other supports in time (see Fig 1). With the common use of remote conferences, those tasks can be expected to lighten in the coming months.

The tasks related to the reception of attendees (i.e., the locker room, the badges and conference bags containing the program, the transportation tickets, lunch and dinner tickets, Wi-Fi code, and the contacts with the conference center staff), as well as meals and coffee breaks logistics, will be cancelled (see Fig 1). Organizing committee members dedicated to these tasks could thus be reassigned to new tasks directly related to the remote conference. This reassignment is a sensitive step. Indeed, people motivated by a face-to-face conference organizational task could lose their motivation if assigned to tasks for the remote format. It is important to take into account the wishes of the organizers. Conversely, a person could get highly motivated by a new challenge of the switch and therefore play a key role in the new organization. After redefining the tasks, you thus have to reassign them based on the new motivations and capabilities of the committee members.

### Rule 4: Select technical solutions

**Fig 2:**
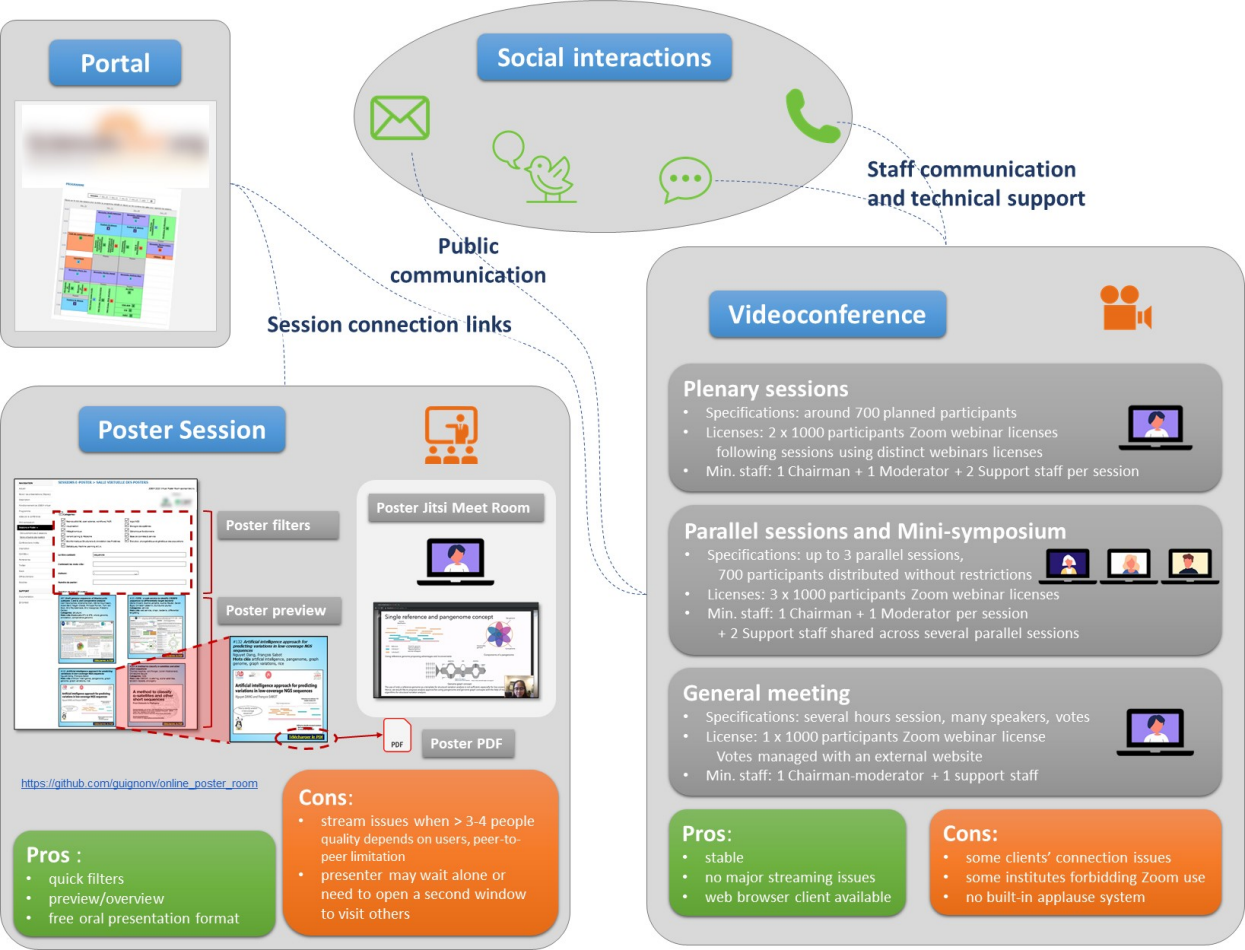
JOBIM 2020 remote conference selected technical solutions and organization. For the plenary and parallel sessions of the conference, but also for our meetings, we selected the Zoom platform (three “1,000 participants webinar” licenses fulfilled our needs). For the poster session, we chose Jitsi Meet and created 1 room for each poster using a bespoke web interface. All the links were available through the conference website (hosted by SciencesConf.org; https://www.sciencesconf.org/). For social interactions, on top of email and phone calls, several platforms were used: Slack, WhatsApp, and Twitter.

There is no silver bullet that fulfills all the needs of any conference. Several tools must be assessed and selected with care. Consequently, you need to choose the best tools according to the conference specificities and initial purposes. First, you will have to identify conference needs and ask the right questions (see S1 Text). Before selecting any technical solution, you will have to start by listing every conference event, from the “welcome” at the information desk to the conference closure and identify the needs behind each of them. Then, you will need to find remote solutions matching each of those needs without focusing on software. Subsequently, available technical solutions should be investigated, compared, tested, and selected. For each choice, keep in mind technical constraints such as the number of attendees and their location, and reconsider parallel sessions and social interactions. For JOBIM 2020, we chose a combination of tools known to be stable, allowing the connection of more than 1,000 attendees (Zoom and Jitsi) and attempting to preserve interactions among attendees as much as possible (Slack, WhatsApp, etc.; see Fig 2).

Based on our experience, you may prefer solutions that are known to be reliable, but also consider backup solutions in case of issues. For instance, most conferencing tools provide the opportunity to record the session for people that could not attend the event. Check the reliability of the selected tools by simulating several scenarios with colleagues at least 1 month before the conference takes place in order to have time to adjust in case of issues.

### Rule 5: Anticipate potential problems

A key point for a successful remote conference is to anticipate issues. Problems will inevitably occur during the conference, and it is therefore essential to anticipate appropriate and efficient solutions, clear guidelines, and tested procedures. Although it is not realistic to identify every possible issue, it is important to consider backup solutions that are not reliant on the same elements and to clearly determine when to use them.

Many issues can occur during face-to-face conferences, both technical and logistical. Switching to a remote conference will not prevent issues from occurring; indeed, most of the face-to-face issues will remain, but new ones will appear. At the same time, a remote conference provides advantages as some adjustments can be implemented quickly to tackle problems that would not be solved so easily during a face-to-face meeting.

Most of the problems can be anticipated and solved by applying procedures. The first most important thing to do is to train all the speakers, chairmen, moderators, and even the attendees to use the selected platform. Several technical checks with the organizers and speakers must be performed days or weeks before the conference. This includes checks for microphone and camera quality, camera orientation, speaker’s placement against light, and network bandwidth. Those checks are usually performed by a professional who also provides training and advice (e.g., turning off any software notifications and phones and making sure no other people will interfere during the presentation with noise or bandwidth).

Guidelines and procedure documents (see S1 Table) must be provided to the organizers and speakers and refined through as many rehearsals as possible. Finally, a few hours before the conference opening, a general open training session should be provided to enable attendees to test their own connection and explain the conference procedures and tools. Such an approach will reduce risks of technical issues for both the panel (speakers, chairmen, and moderators; see Table A in S1 Table) and the attendees.

### Rule 6: Set up a virtual information desk

Conference attendees need to know where and when events will happen during the conference. In a face-to-face conference, this task can be handled by an information desk or a written conference program. The information desk is also the place where one attendee can get help from the conference staff. In the case of a remote conference, these two needs still have to be addressed.

**Fig 3:**
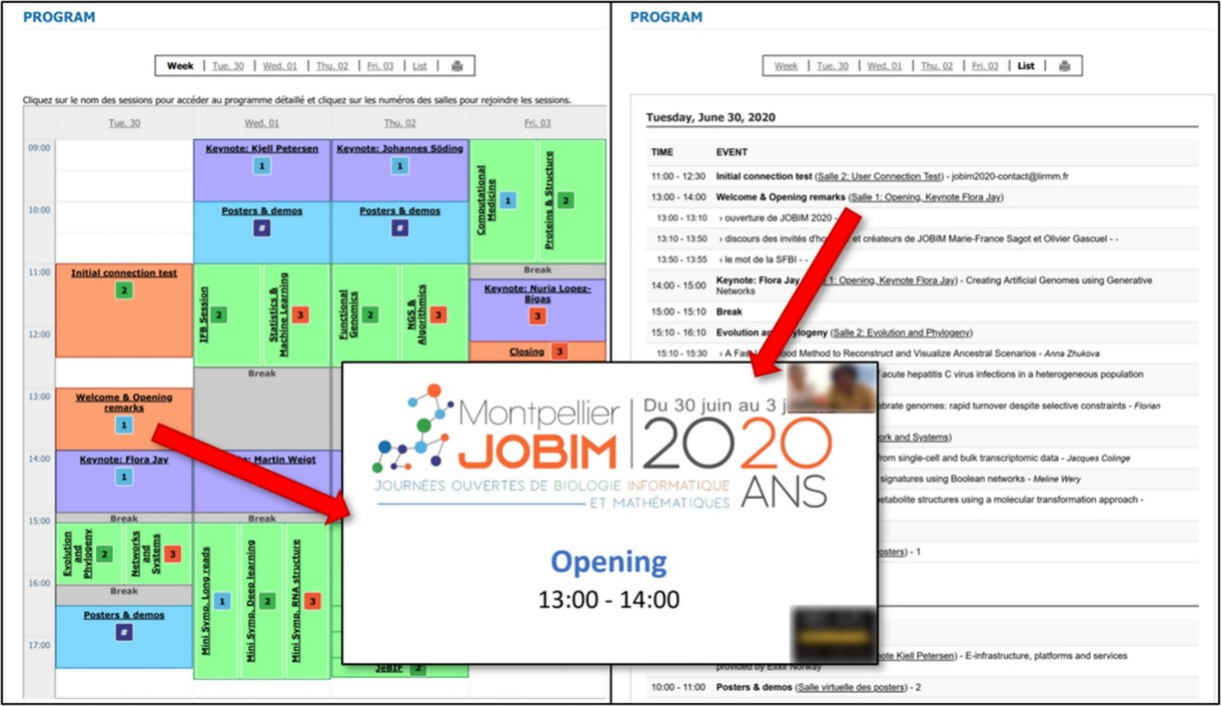
JOBIM 2020 remote conference program in 2 formats. Week view on the left with room links in colored squares and list view on the right with room links between parentheses. Room links lead to a Zoom conference session as shown in the center. The screenshot of the conference program is from a homemade tool we developed specifically for this conference. JOBIM, Journées Ouvertes en Biologie, Informatique et Mathématiques.

The conference program should be made of web pages with event names, times, and locations such as a URL or a tool name with access identifiers (see Fig 3). Those pages can be access restricted if attendees must be registered. The program page format only needs to be intuitive and could include a calendar page, a map of a virtual conference center, a registry, or a combination of those.

Conference help should be provided as an online user guide (see Table A in S1 Table) explaining how to follow rules during the conference and what to do in case of issues. It is also a good idea to include screenshots, an FAQ, and troubleshooting guidelines (see Table B in S1 Table). General information, including the conference website link, could also be summarized in an initial conference welcome email to attendees.

To provide on-demand help, a remote conference must also have a tool where attendees can directly contact support staff. It could be a public email address or a web contact page but, in our case, a live chat system proved to be most efficient. It could be integrated into the conference website, or it could be provided by dedicated chat tools or even an open virtual meeting room. In any case, there should always be some conference staff assigned to this task, ready to answer questions. The most important thing is to choose a reliable tool because if something goes wrong with the videoconference system or the website, people need a place to go for help.

### Rule 7: Preserve social relationships

On top of scientific networking, socializing is another essential part of classical conferences. However, in a remote conference, it is difficult to recreate social interactions outside the conference sessions. Nevertheless, many solutions and approaches are available to preserve social relationships and networking. Some games or challenges before and during the conference can be organized and presented at the end of the conference. As an example, the SFBI organized a game based on members’ photos with the theme “Bioinformatics and lockdown.”

A long-term game to keep people’s attention can be organized, allowing attendees to compete (for instance, through a series of incongruous or geeky words such as “Supercalifragilisticexpialidocious,” “Totoro,” or “42,” e.g., to be identified in the different sessions).

A virtual paper chase game can also be organized before or during the congress (not during the sessions), or you can enable a global chat system on a chat tool during the whole congress. Channels dedicated to technical aspects, job walls, posters, ideas, and random discussions can be set up, and private discussions are allowed, to provide an experience as close as possible to face-to-face events.

Finally, respect the goodies tradition of all congresses, and propose do-it-yourself (DIY) ones, such as folded cups to print on your own, with YouTube tutorials for folding the cups (see Fig 3).

### Rule 8: Be creative and innovative!

**Fig 4:**
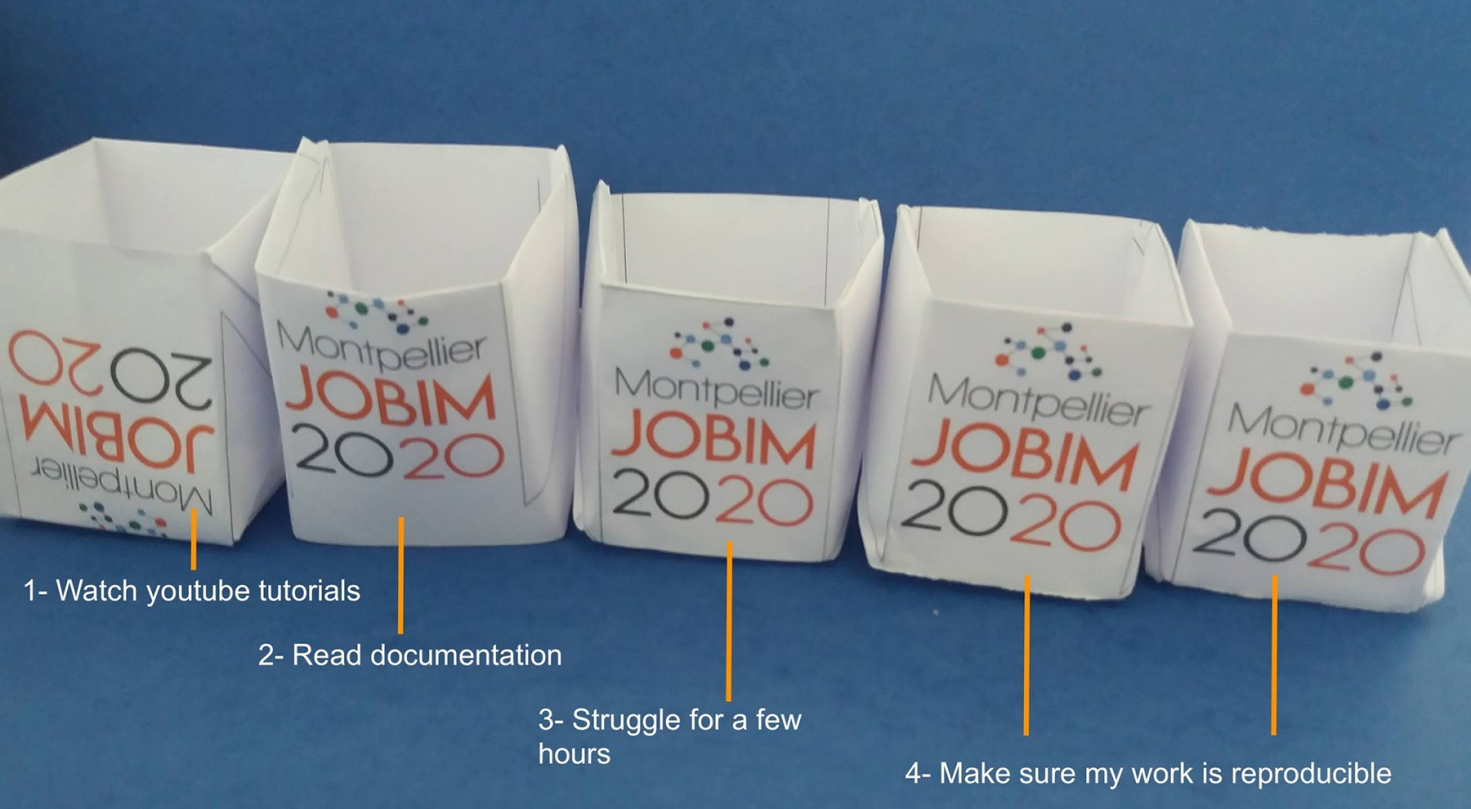
JOBIM 2020 goodies challenge. The original goodies were replaced by a DIY folded cup, with the model to be printed from the website. Participants had fun with this concept and shared their experience with humor and enthusiasm as shown by the legend on this picture. It illustrates well how remote participants were successfully involved in remote social events. *Image credit*: *Nathalie Lehmann*. DIY, do-it-yourself; JOBIM, Journées Ouvertes en Biologie, Informatique et Mathématiques.

It is important to keep attendees motivated and attentive. As technical issues may discourage attendees, the main conference room and the virtual rooms created for the parallel sessions should be easily accessible. The organizers should organize dedicated test sessions before the conference. The technical staff might also provide a phone number for the speakers in case of emergency. The program available on the conference website could also be adapted in order to replace an information desk (see Rule 6 and Fig 3). For international conferences, the program could also be shifted to cover several time zones. Given the numerous time zones, sessions should be recorded and available for all attendees at any time. These videos will also be very important for disable attendees or those with low-quality network. The organizers can imagine special social events such as games or contests before or during the conference (see Rule 7 and Fig 4). The presentation of the winners or the blooper of the contest during the conference would motivate other attendees to play for the next ones. As an example, the SFBI organizes the “best talk award” at each JOBIM conference that was maintained and very appreciated at JOBIM 2020.

**Fig 5:**
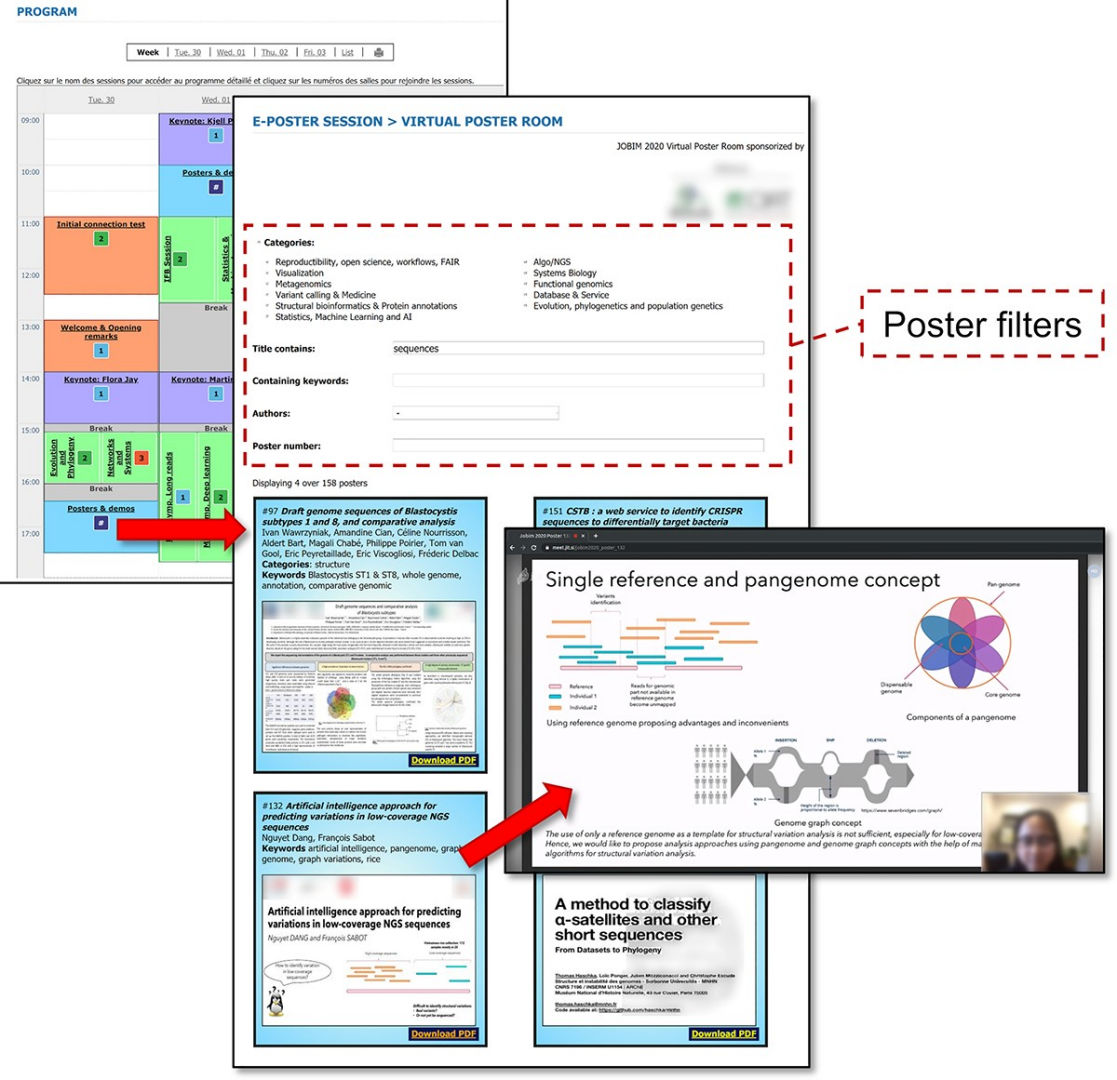
JOBIM 2020 poster session. From the calendar link (top left), the user is brought to the virtual room for posters (at center) [4] where hundreds of posters are displayed and can be dynamically filtered (as framed in dashed red) to only display a list of matching posters. From any poster card, the user can read the summary, have an overview of the poster, download the associated PDF, and reach the Jitsi room during poster sessions. The link to a Jitsi room is unique for each poster and can be displayed only during poster sessions (it is managed by site administrators). *Image credit*: *Nguyet Dang*. The screenshot of the poster session is from a homemade tool (https://github.com/guignonv/online_poster_room). JOBIM, Journées Ouvertes en Biologie, Informatique et Mathématiques.

A conference is a good way to create and establish collaborations, find an internship, or even a job. Exchanges usually happen during coffee breaks, poster sessions, or during the gala evening. Unfortunately, as far as we know, no solution exists to fully replace such exchanges. However, to preserve exchanges as much as possible, the organizers can set up longer time slots for the breaks and other dedicated slots in the program. The poster session should also offer the opportunity to meet and exchange. For JOBIM 2020, we held a poster session with a homemade solution [3] based on the Jitsi Meet platform (https://github.com/guignonv/online_poster_room; see Fig 5). The idea was to dynamically create a room for each poster. After a search on the conference website using poster filtering facilities, the attendees were able to join the poster room and meet the authors or other attendees interested in the topic. To encourage more dynamic and interactive discussions, we also proposed to the authors to present their work through a few slides instead of the classic poster format (A0; see Fig 5). The idea was also to avoid zooming in to view figures or texts. We also invited the authors to prepare a short video of their work, and we selected a few of them to present during the breaks. One can also imagine allowing attendees to schedule meetings for the poster session.

For the poster session, attendees do not want to wait online for an hour without any interaction, and even if we were able to open 2 parallel sessions, it is difficult to go to the other poster to read and discuss with the authors. Platforms such as Gather Town (https://gather.town/) seem to be a solution, because of the possibilities to find the author in the session, meet him/her, and go back near to the poster to present it. Considering the possible technical inequalities between the attendees, it is also important to think about solutions that are as stable as possible, free, but also adapted for people with disabilities. For example, setting up subtitles on recorded talks could allow people with hearing loss to enjoy the conference as much as the other attendees.

### Rule 9: Estimate the reduction in greenhouse gas emissions

As they represent a significant part of academic travel, conferences have to address the problem of sustainability in research. JOBIM 2020 offered a good opportunity to estimate the GHG emissions avoided by switching from a face-to-face to a remote conference.

First, to estimate the emissions of the face-to-face conference, a survey was conducted at registration to know how attendees intended to get to the conference (see S2 Text and S2 Table) and then emissions were estimated using the online application GES 1point5 (i.e., 1.5°C trajectory of the 2019 Intergovernmental Panel on Climate Change (IPCC) report) [5].

**Fig 6:**
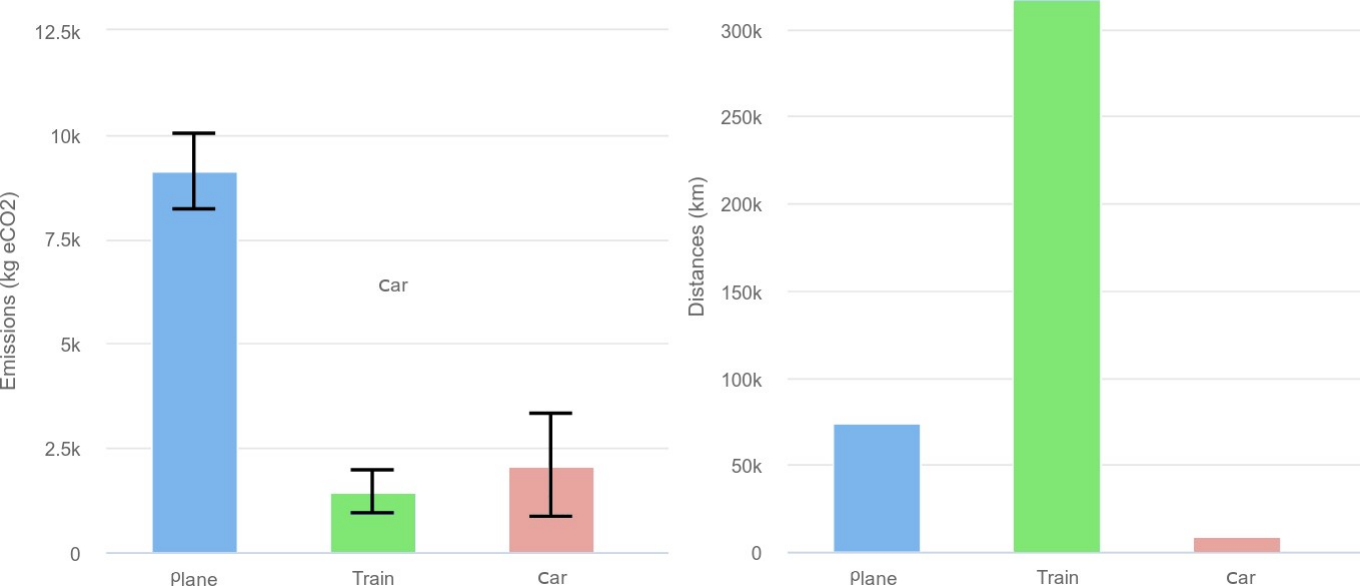
Estimation of GHG emissions and distances traveled. If JOBIM 2020 had been organized face to face, we estimate the GHG emissions and distances traveled, respectively, in kg CO2e and km, using different modes of transport. GHG, greenhouse gas; JOBIM, Journées Ouvertes en Biologie, Informatique et Mathématiques.

Then, estimating the carbon footprint of videoconferences requires determining the emissions of attendees’ terminals, telecommunication networks, and the data center hosting the videoconferencing service, e.g., Zoom servers. S2 Table describes the methodology used. The scope of these 2 estimates is different: Only attendees travels are considered in the face-to-face conference, where GHG emissions of numerical devices, electricity, and video servers are estimated for the remote version of the conference. Even if the GHG emissions of digital usages in a face-to-face conference are not zero, they can only be lower than those of a full remote conference. That’s why we decided to neglect them in the following. Among the 680 attendees registered for JOBIM 2020, 402 said they would have attended the face-to-face conference. Traveled distances and their associated emissions are given in Fig 6. Had JOBIM 2020 been organized in a face-to-face format, the conference would have emitted 12 603 kg CO2e, i.e., 31 kg CO2e per attendee, representing 2% of the maximum carbon budget of 2 tons CO2e [6,7] per attendee to reach the objective of the Paris Climate Agreement.

Among these 402 attendees, only 35 said they would have traveled by plane. Thus, 72% of the total carbon footprint of the conference would have been emitted by 8% of the attendees, while this travel mode represents only 18% of the total distance. These attendees would have had an average emission of 260 kg CO2e, i.e., 13% of the annual carbon budget compatible with the Paris Climate Agreement.

As a remote conference, JOBIM 2020’s total emissions were estimated at 110 kg CO2e, i.e., 0.16 kg eCO2 per attendee, representing 0.01% of the annual carbon budget compatible with the Paris Climate Agreement. These emissions are mainly dominated by manufacturing, transport, and electricity consumption of users’ computers, which represented 93% of the total emissions of the conference (see S2 Text and S2 Table). Considering the previous results, the net emissions avoided by the virtualization of JOBIM 2020 are equal to 12 493 kg CO2e. Although the observed emissions savings are relatively low, they are expected to be significantly larger for international meetings or national conferences taking place in bigger countries than France [8,9,10].

However, it is impossible to talk about digital benefits without addressing rebound effects. Thus, as highlighted in [11,12], the mass use of digital usages would lead to an overall increase in energy consumption due to the manufacture, use and end-of-life treatment of digital devices, and the economic growth generated by digital technologies. These effects might offset digital environmental benefits. Thus, virtual conferences must be part of the solution to reduce carbon emissions by shifting to biennial conferences, selecting the host city based on travel emissions, or creating conference hubs [4].

### Rule 10: Get feedback, learn, and disseminate

Within a continuous improvement approach, it is important to get feedback from attendees. A classic way to get feedback is to ask attendees to fill out a feedback survey. When designing this survey, we decided to kill two birds with one stone: We added questions to get information required to estimate the GHG emissions. Instead of classical catering-related questions, we asked for feedback about the tools, the way the congress was organized, the feelings of the attendees about the connections between the different rooms, and the social interactions through Slack (https://slack.com) or Twitter (https://twitter.com). These results were made available to the community, and other conference organizers have already contacted us to benefit from this feedback in order to optimize their own congress.

Disseminating the lessons learned is an important task that should not be neglected, as more and more conferences will go virtual. At the same time, technologies evolve, people try different things, and new solutions are developed. Sharing the experience of a remote conference, the decisions made, the undertaken strategy, and the tools used will definitely help others to discover and select the appropriate methods to implement their own remote conference. Just like consumer star evaluations on items sold by a website, the feedback on remote conference tools will help both the conference organizers and solution providers to provide better organized remote events with improved solutions.

## Conclusions and future

An important part of a scientist’s job is to disseminate their results during seminars, meetings, and conferences. Since 2020, due to the COVID-19 pandemic, our means of communication and exchange has suddenly and drastically changed. We are living with heavy public health restrictions, which result in a “stop-and-go” organization that compels us to be always ready to change our plans at the last minute.

As the organizers of the annual French bioinformatics conference (JOBIM 2020), we had to face this stressful situation. While numerous conferences planned in 2020 had been cancelled, we decided to maintain the scientific link by switching from a face-to-face to a remote format in less than 3 months. Being one of few remote conferences, a huge amount of work had been achieved to find the best technical solutions. At this time, the best solution was a combination of stable and available tools (e.g., Zoom, Jitsi, and Slack). Since then, the number of new solutions has increased; while some of them attempt to simulate real conference rooms or spaces, others are investing more in optimizing social interactions (e.g., Spatial.Chat or Gather.Town). An important aspect for the computational science community is the suite of open-access solutions that are also emerging (e.g., Mattermost and Rocket). However, the needs may greatly vary based on the number of attendees and the type of conference. Consequently, it may be necessary to optimize or implement complementary features for existing solutions such as for parallel or poster sessions.

The choice or development of these tools must also take into account the inequalities and disabilities of the participants. One solution to overcome such situations and at the same time keep the attendees’ motivation high is to give access to recorded talks after the conference. However, even if several video platforms such as YouTube allow easy sharing of videos, it is crucial not to forget the security of intellectual data and to prefer open-access and academically hosted solutions that respect intellectual property rights.

GHG estimation is an interesting consequence of changing conference formats. For the JOBIM 2020 conference, we estimated the carbon footprint impact to be less than if it had been held face to face. As they represent a significant part of academic travel, conferences are key to raise attendees’ awareness of sustainability, mitigate GHG emissions, and raise the understanding of the impacts of research activities on the environment and the climate.

While waiting for a return to global stability, it is important to maintain scientific links and therefore to maintain scientific events. For now, a good compromise could be to organize local face-to-face hubs or hybrid conferences, i.e., both online and on-site conferences around the country to preserve social relations, while reducing GHG emissions by holding virtual conferences, such as JOBIM 2021 (https://jobim2021.sciencesconf.org/) and SimHydro 2021 (https://www.simhydro.org/).

## Supporting information

S1 Text Nonexhaustive review of questions and some technological answers in 2020. (DOCX)

S2 Text Method used to estimate the carbon footprint of a remote conference. (DOCX)

S1 Table Guideline examples. **Table A:** Chairmen and moderators guidelines used in JOBIM 2020 conference. **Table B:** Speaker guidelines used in JOBIM 2020 conference. JOBIM, Journées Ouvertes en Biologie, Informatique et Mathématiques.
(DOCX)

S2 Table GHG emissions per session. GHG, greenhouse gas. (DOCX)
