# Supplementary material for: Ten simple rules for switching from face-to-face to remote conference: An opportunity to estimate the reduction in GHG emissions
Source: PLoS Comput Biol. 2021 Oct 18;17(10):e1009321. doi: 10.1371/journal.pcbi.1009321 (PMC8523038; doi:10.1371/journal.pcbi.1009321)
Supplement: S1 Text — (DOCX) [file pcbi.1009321.s001.docx]

## **S1 Text**: Non-exhaustive review of questions and some technological answers in 2020.

This appendix provides a non-exhaustive list of questions one could ask before switching from a face-to-face conference to a remote one. It is provided as an example to help conference organizers find the right kind of questions and some technical solutions that could be considered. The reader must keep in mind that the provided solutions were valid at a given time but may be obsolete at the time reading.

**Question:** **What conferencing format will be needed?**

**Purpose:** Meetings are designed for open discussions between a couple of participants.
Livecasting and webinars are designed for few speakers but a large number of attendees.
Videocasting is for video on-demand but not live events.
Instant chat and voting systems enable live textual communication between as many participants as needed.
Virtual Worlds or Virtual Reality Systems display a virtual environment in which participants can move and see other participants for social interactions.
Shared documents and whiteboard systems enable participants to work on the same document and interact through that document.
Forums provide a place for asynchronous and threaded discussions.

**Solutions:** Usually, several solutions are needed and used during a conference.
**Meetings**: The more participants there are, the harder it is to manage discussions. Everybody can show his or her video but usually only one screen (or slide show) can be shared at a time.
To name a few: *Zoom Meeting, Webex, GoToMeeting, Bluejeans Meeting, Jitsi Meet, Microsoft Teams/Skype, Livestorm Meeting,...***Webinars**: Only presenters videos or screens can be displayed and attendees watch and listen. There are often 2 sides: the presenter side which behaves just like a meeting and the attendee side focused on watching, listening. Webinars are designed for a large audience (ie. larger than meetings) while attendees may be allowed to talk and share their video by moderators when needed. Attendees often have the possibility to also interact by raising a virtual hand or through a chat or through a question and answer system or even a voting system.
To name a few: *Zoom Webinar, GoToWebinar, Bluejeans Events, Livestorm Webinar,...***Livecasting**: It is a webcast that is streamed live usually for a very large audience (ie. larger than webinars). It is not designed for interactions with the audience like webinars but often provide a chat system to manage interactions at minima. It reduces social interactions but allows a larger audience and usually only requires a web browser on the client side.
To name a few: *YouTube Live, Vimeo live events,...*
**Videocasting** (vidcast, vodcast):It intends to stream video on demand.
Therefore, it does not intend to (but it could) provide live videos but is suitable when attendees’ time zones are an issue. It usually supports a large audience like livecastsing.
To name a few: YouTube, Mediasite, Wowza Streaming Engine, Dacast,...
**Chat rooms:** They can be split in chat rooms or threads and used for attendees reactions during a talk, for discussions on a topic, for questions, for technical support, for advertisement and other types of textual communications.
To name a few: *Slack, Discord, IRC servers, website live chat software,.*..
**Voting systems:** They are suited to manage attendees votes or questions. They can offer anonymization, enables live votes and questions and sometimes allows questions to be voted up.
To name a few: *Zoom Webinar (when questions are enabled), Slido,.*..
**Virtual Worlds** or Virtual Reality Systems: They can be used to solve some aspects of the social relationships missing in a webinar or a remote meeting, like coffee breaks or even poster sessions. They provide the opportunity to have unplanned meetings between participants and informal talks. People can see who is present and look for collaborators to discuss with. However, they often hardly support a large number of attendees.
To name a few: *Gather.town, Mozilla Hubs, OpenSimulator*,...
**Shared documents and whiteboard systems**: They can be used both for work collaboration (during breaks or workshops), public display (offers, advertisement) and public interaction (with a small number of attendees to make them participate actively during presentation for instance).
To name a few: *Google docs, Microsoft SharePoint, HackMD, whiteboards integrated into meeting softwares*,...
**Forums:** It is an asynchronous way to collect people's reactions separated into threads (ie. adapted to multiple time zones). It could be seen as an alternative to chat rooms that are not “live” and with larger messages.

**Question:** **What will be the maximum number of attendees?**

**Purpose:** It will condition the video conferencing software selection. The more attendees, the fewer choices.

**Solutions:** Each type of event will have different limits: meetings usually support less participants than a webinar and remote poster sessions or virtual coffee breaks really depend on the format wanted.

**Question:** **Will attendees share time zones?**

**Purpose:** While a face-to-face conference was supposed to occur at one place in a definite time zone, a remote conference will see its participants attend from where they live. For international conferences, it brings the issue of time zones as live events could occur when some attendees are usually sleeping.

**Solutions:** Several solutions can be considered: stick to the original face-to-face time zone and provide replays (video-on-demand) or group time zones and repeat the live events several times (two times are often enough) so each group of time zone has an opportunity to participate in each live event. Prefer asynchronous tools.

**Question:** **Where will the attendees be most likely located?**

**Purpose:** Knowing attendees’ location may be relevant for bandwidth issues. Audio-video streaming consumes a large bandwidth and streaming servers should be located as close as possible as the largest number of attendees.

**Solutions:** When choosing a video conferencing service provider, take into account where its servers are located.

**Question:** **Will conference access be restricted (paid)?**

**Purpose:** If accesses have to be restricted, it will impact tools selection as only few of them handle well permissions. Most tools just rely on a single password or a secret URL that can be shared with unallowed users. It may become a real issue at live time if those tools are not designed or not licensed to support more clients than the registered ones. For instance, if a paying webinar is limited to 100 participants and its secret URL is shared with unregistered users, users who registered and paid their fees may not be able to join the conference if the limit is reached (by unregistered users) before they arrive.

**Solutions:** If the selected tool only supports a global password for all attendees, do not use an easy to guess password. Also consider changing the password between sessions and provide it just a few hours or days before the event. However, changing the password will confuse attendees. Some video conferencing systems automate sending access mails to attendees at a given time chosen by the administrator. Another way would be to use the conference website as a login portal from which registered attendees will get the correct link just a few minutes before the event starts but the website administrator will have to ensure all attendees are registered and do not share their credentials. Also, at the expense of a lot of efforts, some video conferencing providers allow attendees to login through third party websites from which accesses are managed (for instance Zoom vanity URL). Finally, it might be wiser to not spend too much effort in securing access to the conference and rather get prepared to support more attendees than expected and have well trained staff who can quickly exclude and ban bad attendees that may cause trouble.

**Question:** **Will there be several webinars or meetings running at the same time ?**

**Purpose:** Usually, video conferencing providers afford licenses limited to a given number of participants. If parallel sessions are needed, attendees may not split equally between sessions and each session may require a license supporting the maximum number of participants. For instance, if a conference has 100 attendees and two parallel sessions, each session may require a 100 attendees license because one session may be more attractive than the other or for some reason, one could be canceled but not the other.

**Solutions:** Try to estimate or guess the size needed for each parallel session but keep in mind that all attendees may choose the same session. Also consider that paying for two (or more) licenses of the maximum number of attendees can also be useful if the next conference should be started (maybe just in training mode) before the previous one ends. Having an extra-license is worth it especially when considering its cost compared to the one of an extra “real” room rental.

**Question:** **Will sessions be recorded? For which purpose? Available for how long?**

**Purpose:** Session recording implies several elements: either the video conferencing tool allows it natively or a third party tool is required; speakers agreed to be recorded as well as other participants; depending on the recording definition and the number and the length of sessions, a lot of storing space will be required. Then, the records may be served on demand and it should also be considered.

**Solutions:** It can be achieved through service providers or internally with a dedicated server. A videocasting system should be selected with care: which available bandwidth for how many clients? How to secure recordings (backups)? Access restrictions? Availability ? It must be anticipated.

**Question:** **How questions will be handled?**

**Purpose:** A conference without support for attendees’ questions is not a proper conference. One way to handle attendees’ questions must be set up.

**Solutions:** Many solutions are available from the less convivial such as forums (adapted for asynchronous talks) to the more advanced ones such as votable question lists where attendees can both submit written questions and vote for them. For more human interactions during live events, we recommend that a chairman picks up questions from a question-submission-and-voting system and reads it out loud to all while the attendee who submitted the question gets the permission to talk and complete its question by oral if needed.

**Question:** **Will there be satellite meetings and needs for a voting system?**

**Purpose:** Sometimes, conferences are the right place to have parallel meetings for projects, or scientific assemblies as most concerned people are attending the conference. If scientific assemblies are taking place during the conference, they may require an electronic voting system (for elections for instance) to replace show of hands.

**Solutions:** Either remote meeting rooms can be licensed and provided on demand to attendees, or attendees can manage themselves their own meetings. For electronic votes, many third-party solutions are available as well as homemade ones but, in any case, participants will have to be notified of the process several days before it takes place.

**Question:** **Will there be a poster session?**

**Purpose:** If there is a poster session, what are its expectations? Is it just to present work without interactions? Is it for networking? Should there be questions and answers? Live? Usually, poster sessions are there to allow communication on a large number of subjects in parallel in limited time. Attendees know the list of posters in advance and often have a selection of posters they want to visit. Sometimes they just want to walk though posters and stop in front of posters of interest. Having a poster presenter next to it allows social interactions, scientific discussions and networking, and it is a real opportunity for young researchers to present their work.

**Solutions:** To fulfill those face-to-face conference needs with a remote one, there may be a virtual poster gallery. It could be a virtual 3D gallery or just a simple webpage with the list of posters. However, contrary to a face-to-face conference, a virtual poster session offers some advantages: posters of interest can be easily filtered and found, and poster presenters can present with more materials than just a poster PDF. They could provide a live or pre-recorded presentation on demand but also perform live demos (screen sharing). Guidelines to poster presenters should be provided weeks before the session in order to let them get prepared.
In the case of JOBIM 2020, we used a poster virtual room (webpage) that provided poster filtering facilities, previews and links to Jitsi Meet rooms (one per posters) since Jitsi Meet rooms are not using a central streaming server but rather direct client-to-client connections. It works well with a limited number of attendees on a poster but gets complicated with more than four clients and even worse with poor network connections. Another drawback is that the poster presenter could wait without seeing anybody during the whole poster session. Therefore, we allowed presenters to keep their room on but empty, visit others at the same time and come back to their room if somebody gets connected. The success of this approach was mitigated. If we had to do it again, we would try a different approach relying on virtual worlds like Gather.Town to improve social interactions. However, we do not know how such systems would behave with several hundreds of participants and if we would have to spread posters across several rooms.

**Question:** **Will there be training, workshops or demos?**

**Purpose:** For such events, going remote will offer new opportunities. Every participant is already in front of a computer with an internet connection. There will be no restrictions on the number of rooms available nor computers. However, computers would not be pre-configured with the appropriate softwares.

**Solutions:** Two approaches can be considered. The first one would be to let each presenter provide a list of requirements that will be published on the conference website days before the conference to let time for attendees to set up the appropriate environment if they want to attempt a given session. The second approach could be to provide some minimal requirements like installing Docker or a virtual machine on the attendee’s computer for any session and every presenter will provide live instructions using what has been installed.

**Question:** **Will there be remote coffee breaks?**

**Purpose:** Coffee breaks could be neglected but they are also an important part of the conference: it is the place for social networking. It is easier to imagine how to virtualize a seminar or a meeting than a coffee break. However, it is not impossible and maybe even as important as webinars and meetings.

**Solutions:** The most basic way of implementing a coffee break would be to use chat rooms (or forums for asynchronous conferences). However, not many people will connect to the chat room and wait for something to happen or often check the whole list of attendees to find someone they know. It is also frustrating to try to chat with someone and have no answer without knowing if it is because the person is not there or because he or she is already talking with someone else. In the opposite, it is very hard for someone popular to answer many people at the same time in different conversations. In that sense, virtual worlds reproduce quite well what happens in real life as one can see who is there and who is talking to who. Furthermore, it is more entertaining to visit a virtual world even if you meet nobody you know, than waiting in a chat room.
In the case of JOBIM 2020, we regret we did not have the time to find, investigate and set up solutions like Gather.Town for instance. The playful aspect of such solutions would have certainly attracted people and led to successful virtual coffee breaks or even social diner.
