# Supplementary material for: Ten simple rules for switching from face-to-face to remote conference: An opportunity to estimate the reduction in GHG emissions
Source: PLoS Comput Biol. 2021 Oct 18;17(10):e1009321. doi: 10.1371/journal.pcbi.1009321 (PMC8523038; doi:10.1371/journal.pcbi.1009321)
Supplement: S2 Text — (DOCX) [file pcbi.1009321.s002.docx]

## Method used to estimate the carbon footprint of a remote conference.

Estimating the carbon footprint of a remote conference is complex and remains an active ​​research area. This estimation depends on three sources of emissions: the telecommunication network used to transfer data, the data center where the remote conferencing service is hosted and terminals used by attendees. The carbon footprint, denoted $CF$, of the $S$ virtual conference sessions of JOBIM is given by

$CF =\sum_{s=0}^{S} P_{s}T_{s} (F_{n}+F_{d}+F_{t}),$

Where $P_{s}$ and $T_{s}$ respectively represent the number of attendees and the duration of the session $s$ and where $F_{n}$, $F_{d}$ and $F_{t}$ respectively correspond to the average emissions factors of the telecommunications network, the data center and attendees terminals. These three factors are defined in the following sections and are expressed in kg CO2e per minute.

***Telecommunication network carbon footprint***. To date, few studies allow the integration of carbon emissions linked to data transport. The only available model to our knowledge is the 1byte model [5] of the Shift Project. The first step of this model consists of converting into kWh the quantities of data transferred during the remote conference. This energy consumption depends on the network used, *i.e.* cable, WIFI or 4G. In the context of JOBIM, the assumption made is that the cable network was mostly used, coming with a factor of 4.29e-10 kWh per Byte transferred. The second step of the 1byte model refine the estimation according to the energy mix of the country. As the network is global, the emission factor used is the average world electricity production factor, *i.e.*, 0.519 kg eCO2 per kWh [7]. To determine the bandwidth consumption of JOBIM conference sessions, 30 ten minutes measurements were recorded on two different terminals using nethogs. The average data transfer obtained is 506 kB ± 86 kB. This allows to determine the average emission factor of the telecommunication network, $F_{n}= 4.29e^{-10} * 0.519 * 50.6e^{3} = 1.13e^{-5}$kg CO2e per minute.

***Data center carbon footprint.*** In the same way as the network footprint, the 1byte model [5] of the Shift Project offers a factor of consumption per amount of data transmitted by a data center. This factor of 7.20e −11 kWh per Byte transferred gives $F_{d}=7.20e^{-11} * 0.519 * 50.6e^{3} = 1.89e^{-6}$kg CO2e per minute using the factor emission of the average world electricity production and bandwidth consumption during the conference.

***Attendees terminals carbon footprint.*** According to the Ecodiag database version 2020-11-18-2813ba2 [7], developed by EcoInfo, the emission factors, including manufacturing, transport and electricity consumption, are different from one terminal to another, *i.e.*, 183 kg CO2e per year for a stationary computer with its screen, 69 kg CO2e per year for a laptop, 42 kg CO2e per year for a smartphone and 75 kg CO2e per year for a tablet. To determine an average emission factor, a survey was carried out at the end of the conference to know the proportion of each terminal used. Of the 190 answers, 133 attendees used a laptop, 51 a desktop computer, five watched the conferences on a smartphone and one used a tablet. This distribution allows to define $F_{t}=1.89e^{-4}$kg CO2e per minute per terminal.
