# Supplementary material for: Ten simple rules for switching from face-to-face to remote conference: An opportunity to estimate the reduction in GHG emissions
Source: PLoS Comput Biol. 2021 Oct 18;17(10):e1009321. doi: 10.1371/journal.pcbi.1009321 (PMC8523038; doi:10.1371/journal.pcbi.1009321)
Supplement: S1 Table — Table A: Chairmen and moderators guidelines used in JOBIM 2020 conference. Table B: Speaker guidelines used in JOBIM 2020 conference. JOBIM, Journées Ouvertes en Biologie, Informatique et Mathématiques. (DOCX) [file pcbi.1009321.s003.docx]

## **S1 Table**: Guideline examples.

The following guidelines were generated as PDF files and shared with all moderators, chairmen and speakers. They were improved during training and used during the conference.

# **Table** A: Chairmen and moderators guidelines used in JOBIM 2020 conference.

## Division of roles

The moderator (**MOD**) is the virtual microphone carrier. They manage Zoom webinar features, but they should not have to talk. To summarize:

- Manage other microphones: mute or ask for microphone activation for chairmen, speakers, and attendees.
- Manage screen sharing: provide the default conference slides with current conference program and get back screen sharing when a speaker has finished.
- Moderate chat and questions: remove inappropriate content and ban unwanted participants.
- Provide help on basic technical issues.

Chairman/chairwoman (**CM**) behave almost just like in face-to-face meetings:

- Introduce a session.
- Introduce speakers.
- Manage session time.
- Manage questions and answers (Q&A).
- Thanks speakers and the audience.

Other roles are speakers (**SP**), attendees (**AT**), attendees who asked a question (**ATq**) and Relay Webcasting (**RW**), our technical support provider.

## Conference session phases

| **v1.5** | **Moderator (MOD)** | **Chairman/woman (CM)** |
| --- | --- | --- |
|  | 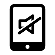 Phones in sleep mode  Turn off notifications (e-mail, skype, etc.)  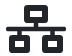 No internet line sharing  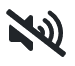 No neighborhood noises  Slack running on JOBIM 2020 channel #general  Zoom installed on smartphone (for computer loss optional) | |
| 1) Practice phase: **at least 10 min before** session start | Open webinar session (practice mode, no attendees yet)  URL:<https://www.zoom.us/>  Select zoom account according to the webinar room:  jobim2020-zoom**1**@....fr  jobim2020-zoom**2**@....fr  jobim2020-zoom**3**@....fr  Password: *received by email*  "Start" button on zoom web page:  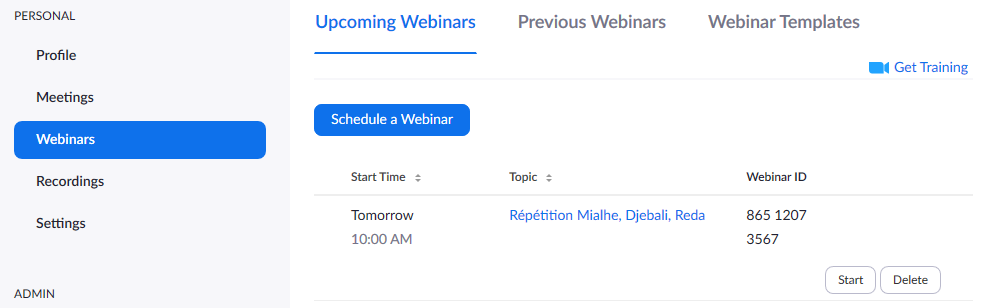  Practice mode (orange stripe on top):  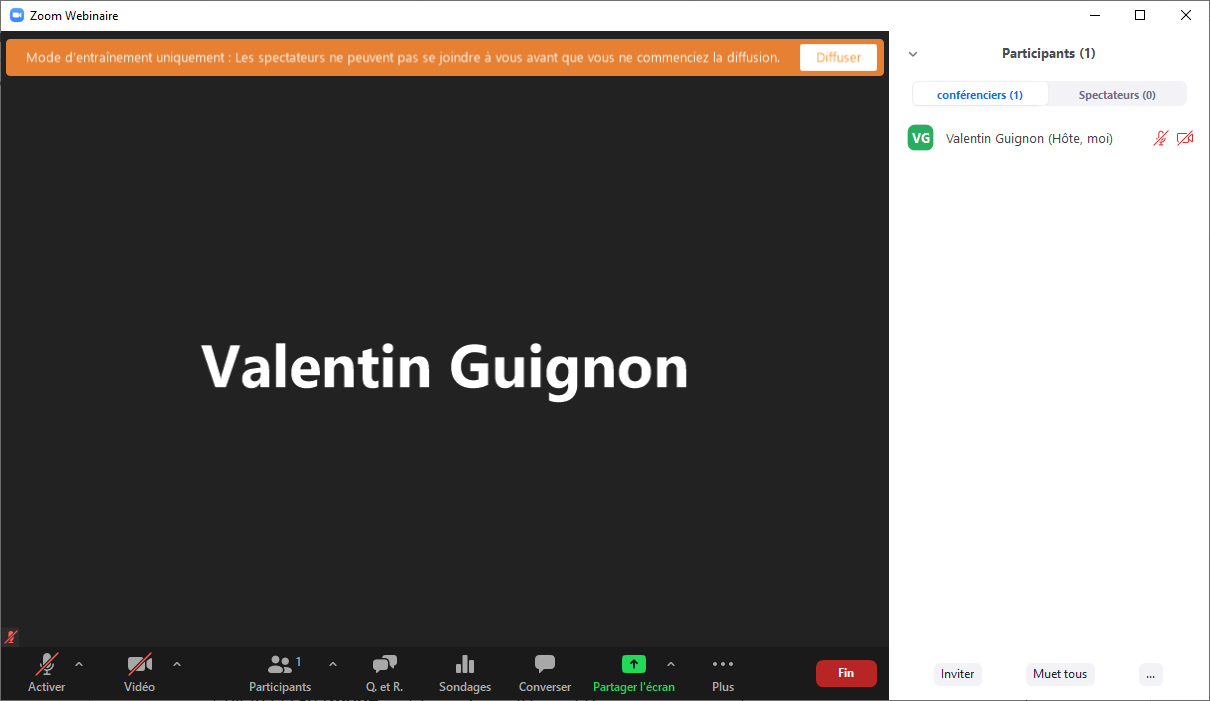 | Connect before attendees in practice mode using link "Click here to join" provided by **email**, which URL contains token "...?**tk=**xxxx..." which avoids getting locked in waiting room  (if sent to waiting room or prompted for password disconnect from zoom application and website, clear zoom cookies, verify used link and try again)  nb.: each session has a unique connection link which is private and customized for you, do not share.  Training mode web client (white stripe on top):  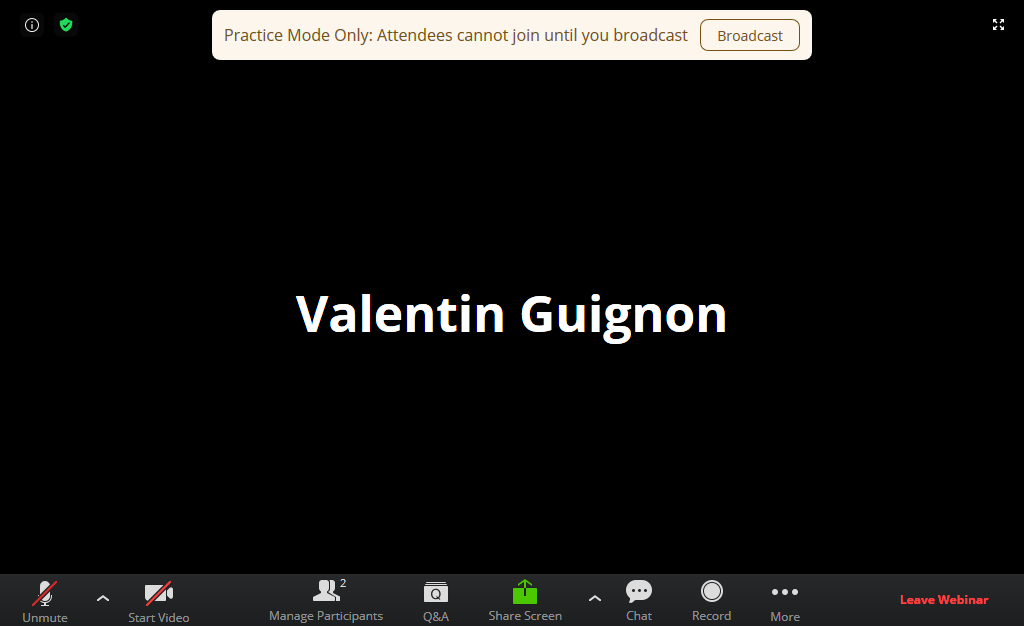 |
|  | Rename yourself from panelist ("**Plus>**"):  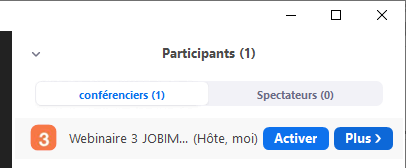  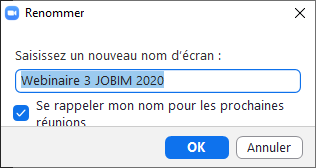  Use format "*First-name Last-name*" |  |
|  | Promote (co-hosts): **MOD**, **CM** and **RW** if not already.  Display panelists: 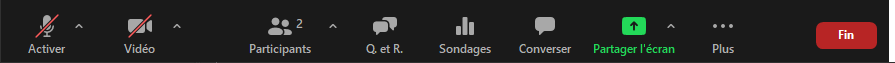  Promote: 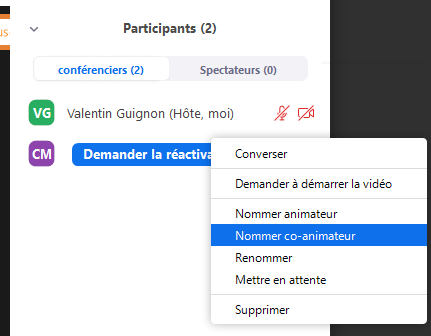  nb: with muted participant, the “promote” button may be hidden on the right. Try to click on the little piece visible as show below or enlarge your window.  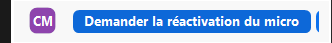 |  |
|  | Share JOBIM session slide   1. Run PowerPoint in show mode, PDF in full screen or as you prefer:  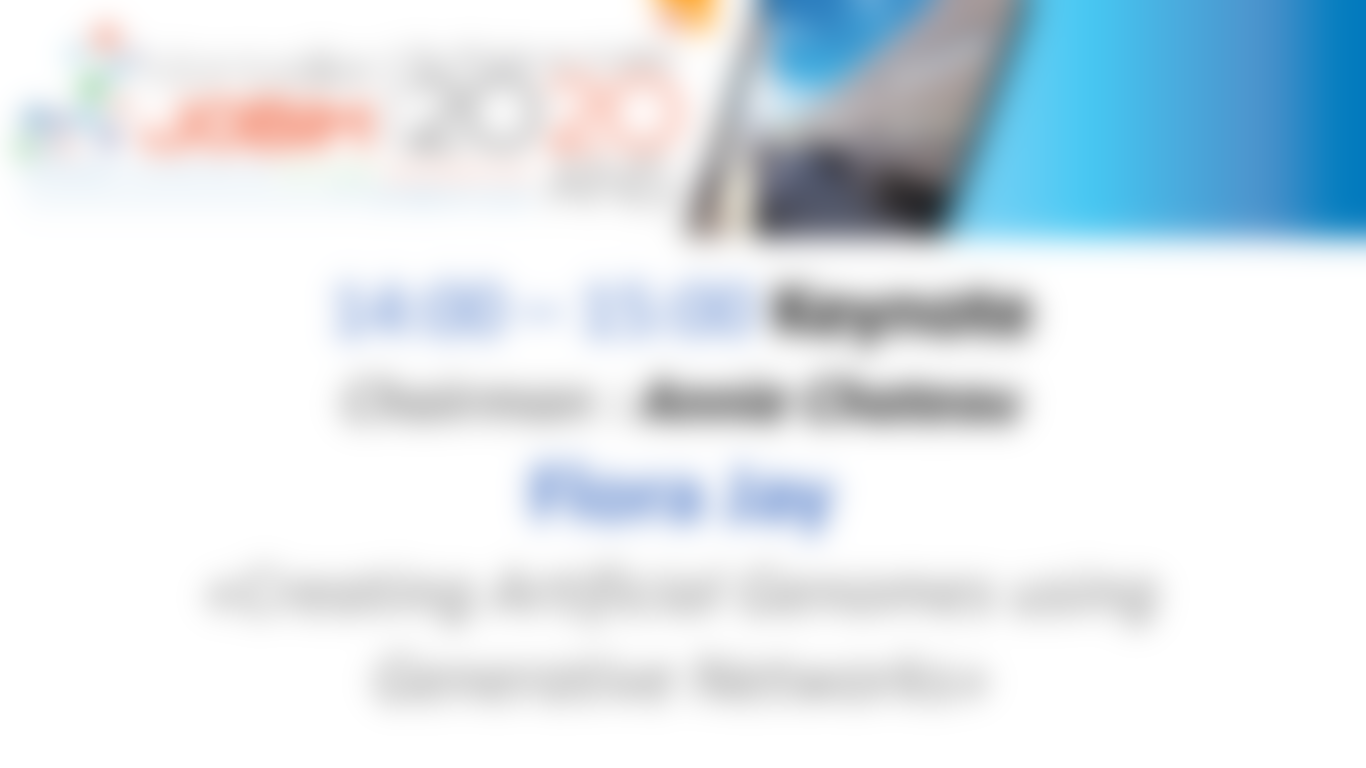  1. Start sharing the slide on Zoom. 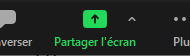 2. check sharing setting using the "**^**" button above: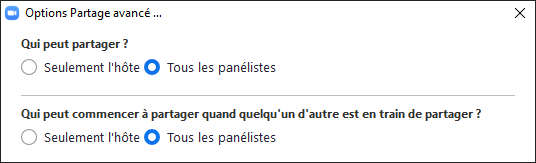 3. check anonymous and question vote settings: 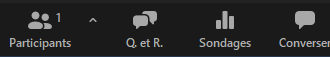 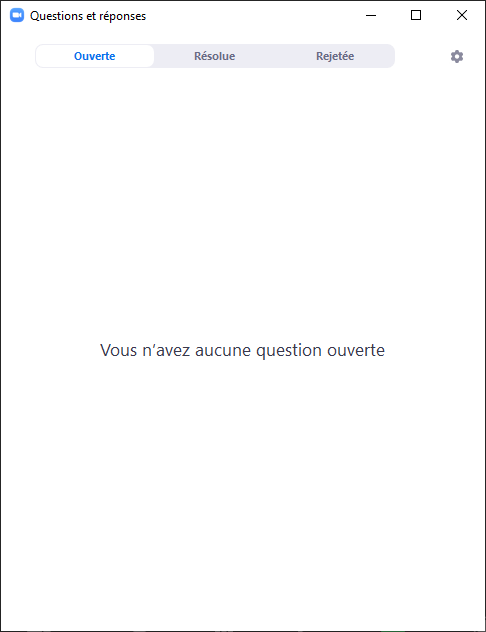 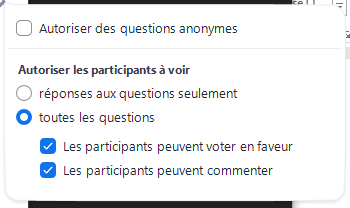 Adjust settings to follow the above pictures. 4. Allow attendees to see each-other: use the “...” at bottom of the attendees list 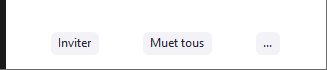   Configure like this: 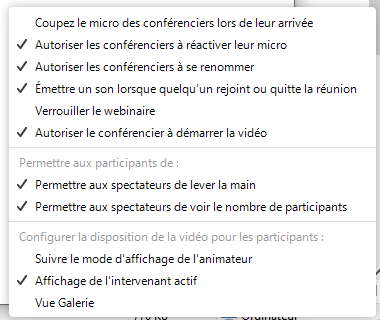 |  |
| All speakers should be in the panel list.  **SP** should display their PowerPoint /PDF in show mode, full screen or whatever wanted to prevent bad window sharing | Check everybody’s microphone and video:  "**MOD***: hello everyone, we are about to proceed to the last checks. M. Chairman, could you enable your microphone and speak please?*  **CM***: yes, hello!*  **MOD***: good. Mrs Speaker could you enable your microphone and speak please?*  **SP***: Hello!*  **MOD***: thanks! Now could you start sharing your presentation?*  [**SP** starts sharing]  **MOD***: OK* [**MOD** take back screen sharing]  *…* [and so on]" |  |
|  |  | **CM** can now chat with **SP** how they would like to manage questions and other aspects of the talk (time notifications for instance). |
| **2-3min before** session starts | **MOD** asks if everybody is ready, closes all microphones but **CM**, next **SP** ones and its own (for countdown). |  |
|  | **MOD** warns for the session opening and reminds to wait a few minutes and to not stop screen sharing and just wait for others to take over screen sharing. **MOD** also reminds **SP** to start sharing while **CM** is finishing to introduce **SP**. **MOD** counts down and opens the webinar (on air) on time:  "**MOD**: *Warning, the webinar session will be opened in 5..4..3..2..1!*"  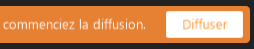  nb: JOBIM slide continues to be displayed | **CM** gets ready to welcome everybody at the session. |
| We wait **around 2min** to let attendees join and start recording. |  | **CM** can explain we are waiting for others to connect. |
| 2) Oral presentation phase | **SP** must “take over” screen sharing, and **MOD** checks **SP** microphone is on. | **CM** welcomes everybody, reminds webinar rules, introduce the session, and introduce **SP** |
|  |  | **CM** checks **SP** took over screen sharing or reminds it, and then lets **SP** talk. |
|  |  | **CM** turns off its microphone and keeps an eye on the chat, questions, and timing. |
|  |  | **1min** (parallel sessions) or **5min** (plenary) before the end, **CM** annotates screen with “*x minutes left*” (bottom right) if **SP** seems late.  "Annotate" button on Zoom bottom bar: 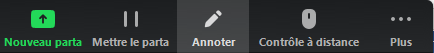  Check size style using "format" button: 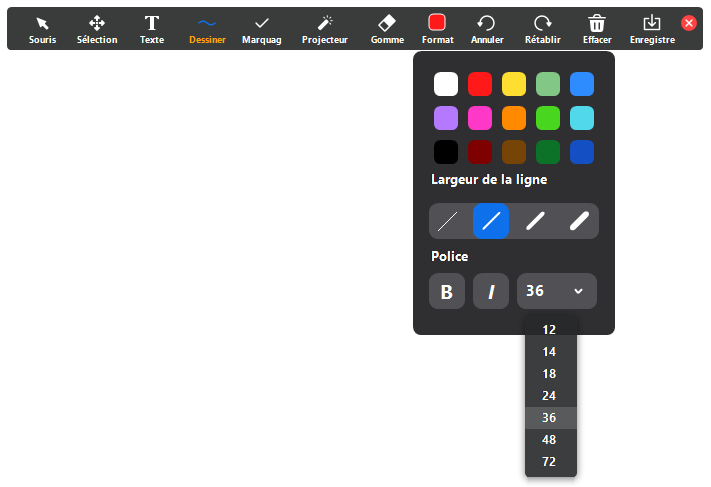  Click on "T Text" button and click in a corner of the presentation screen to enter some text (not visible while typing, avoid special characters).  Click somewhere else on the presentation screen so the text will be displayed, wait a few seconds, and then hit Ctrl+Z or use the eraser button to clear the text. Close annotation bar. |
|  | nb.: no takeover of screen sharing before questions end. | **CM** enables his/her microphone, thanks **SP** and launches the question phase. |

| 3) Question phase | **MOD** finds and enable microphone of the **ATq** on demand if **ATq** raised his hand  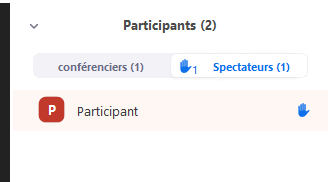  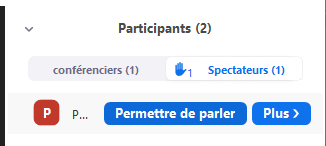 | **CM** names the next **ATq**, and asks **ATq** to raise the hand if he/she would like to talk and **CM** reads the question meanwhile:  "**CM**: *We have a question from M. AttendeeQ; if you would like to talk, use the raise-the-hand button. The question was: How would you why...?*"  **CM** checks if **ATq** has the microphone enabled and if yes let him/her talk: "**CM**: *Would you like to add something to complete or clarify your question?*" |
| --- | --- | --- |
| **SP** answers and the answer is validated by **ATq** or **CM** | **MOD** lowers the hand and closes the microphone of **ATq**.  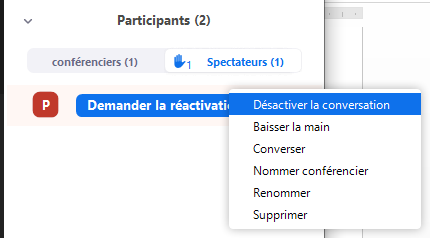  nb: it is the second blue button sometimes hidden. | **CM** checks if there is still some time left and eventually continues the question phase. |
| 4) End of presentation phase | **MOD** takes over screen sharing to display the JOBIM slide of next session. | **CM** thanks again **SP** and the attendees and if needed, reminds for webinar room change. |

### Zoom account connection operating method

[*confidential and not published here*]

### Operating method to play a pre-recorded video

1. Start video player in pause mode.
2. Do not display the video player in full screen and keep original video size.
3. Hide as much as possible the control bar (Ctrl+H for VLC under Windows).
4. On Zoom use the screen sharing button

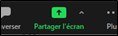

5. Select the two option (dialog window bottom): "**Share sound**" and "**Optimize for full screen video clip**" as show here:

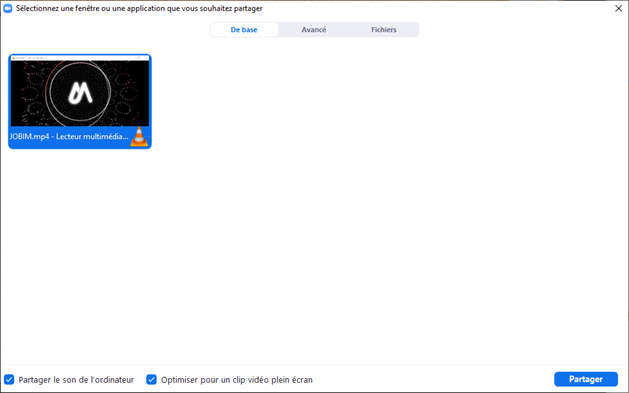


### Troubleshooting

- Practice mode gone in air mode too early: too bad, there is nothing to be done, just keep going with the attendees; do not stop or restart webinar! Maybe explain the issue to attendees.
- **MOD** lost: **CM** takes over **MOD** role while waiting for another solution (provided by support staff)
- **CM** lost: **MOD** takes over **CM** role while waiting for another solution (provided by support staff)
- **SP** lost: his/her PPT/PDF presentation will be displayed by **MOD** at the current page while support staff finds a solution by phone.
- **SP** poor bandwidth/low video stream quality: support staff stops SP camera to save bandwidth. If not enough, the PPT/PDF presentation will be shared and managed by **MOD** (**SP** will ask for next slides).
- Microphone issue: RW will be contacted to solve the issue.
- Video camera issue: RW will be contacted to solve the issue.
- Screen sharing issue: RW will be contacted to solve the issue.
- Zoom connection issue: instructions will be displayed on JOBIM website as well as on the slack channels.
- **AT** reporting issues: **RW** or other support staff may help to solve the issues

In case of Zoom loss: Slack channel JOBIM2020 #general

Link: https://jobim2020.slack.com/archives/CCV6M01T2

**Relais Webcasting** contact:

Mathieu Lahargou

Relais Webcasting

Email: [*confidential*]

Phone or WhatsApp: [*confidential*]

**JOBIM** support contact:

Valentin Guignon

Email: [*confidential*]

Phone or WhatsApp: [*confidential*]

Skype: [*confidential*]

Teams: [*confidential*]

# **Table B**: Speaker guidelines used in JOBIM 2020 conference.

## Division of roles

The moderator (**MOD**) is the virtual microphone carrier. They manage Zoom webinar features, but they should not have to talk. To summarize:

- Manage other microphones: mute or ask for microphone activation for chairmen, speakers, and attendees.
- Manage screen sharing: provide the default conference slides with current conference program and get back screen sharing when a speaker has finished.
- Moderate chat and questions: remove inappropriate content and ban unwanted participants.
- Provide help on basic technical issues.

Chairman/chairwoman (**CM**) behave almost just like in face-to-face meetings:

- Introduce a session.
- Introduce speakers.
- Manage session time.
- Manage questions and answers (Q&A).
- Thanks speakers and the audience.

Other roles are speakers (**SP**), attendees (**AT**), attendees who asked a question (**ATq**) and Relay Webcasting (**RW**), our technical support provider.

## Conference session phases

| **v1.5** | **Speaker (SP)** |
| --- | --- |
|  | 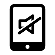 Phones in sleep mode  Turn off notifications (e-mail, skype, etc.)  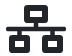 No internet line sharing  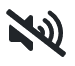 No neighborhood noises  Zoom installed on smartphone (for computer loss optional) |
| 1) Practice phase: **at least 10 min before** session start | Connect before attendees in practice mode using link "Click here to join" provided by **email**, which URL contains token "...?**tk=**xxxx..." which avoids getting locked in waiting room.  There will be a mail with a different link for each session if you speak in more than one and the email sender will be "*Organisation JOBIM 2020*" or from *jobim2020-zoom#-request@lirmm.fr; on behalf of; Organisation JOBIM 2020* and in both cases, "*no-reply@zoom.us*" is used. Check your spam and use the appropriate link to join.  (if sent to waiting room or prompted for password disconnect from zoom application and website, clear zoom cookies, verify used link and try again)  nb.: each session has a unique connection link which is private and customized for you, do not share.  Training mode web client (white stripe on top):  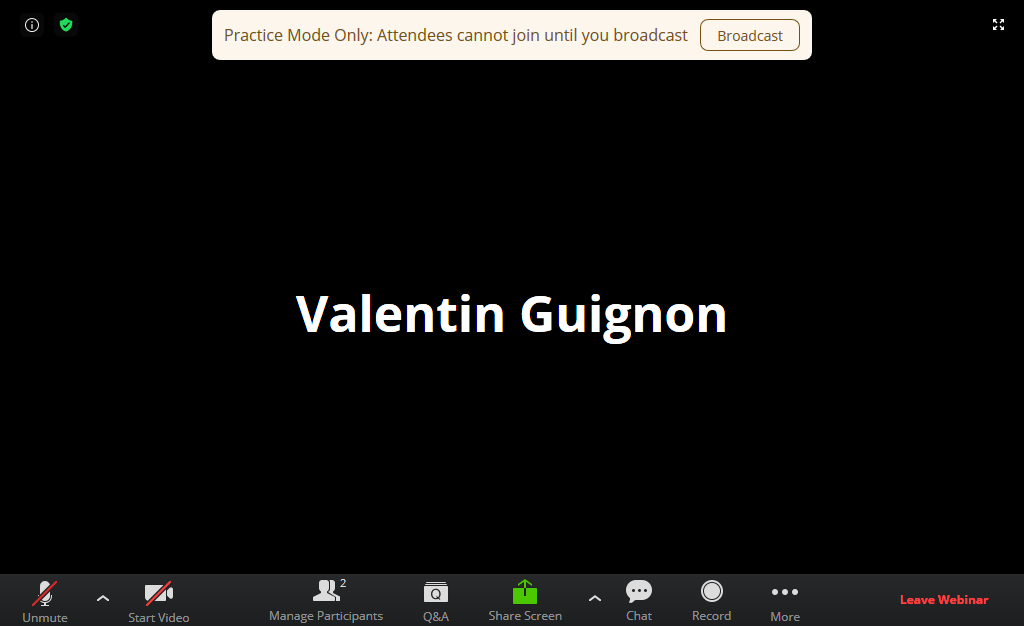 |
|  | Prepare your PPT/PDF presentation and display it in show mode (full screen or whatever suits you better).  Always share a window and not a full screen.  If you share a video, do not display it in full screen but rather keep its original size and use the 2 Zoom screen sharing options (sharing popup bottom) “Share sound” and “Optimize for full screen video clip” as shown below: 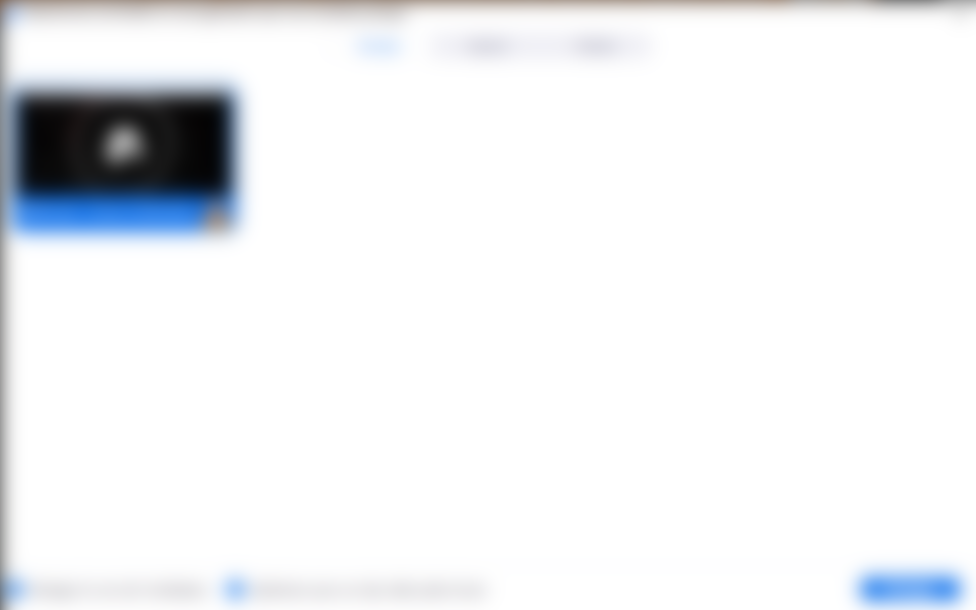 |
| All speakers should be in the panel list | **MOD** should ask you to test your microphone and your presentation slides.  Start sharing your presentation on demand.  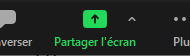  Do not stop screen sharing and just wait (or ask) for **MOD** to take over it. |
|  | Check with **CM** how you would like questions to be handled and other aspects of your talk (time notifications for instance). |
| **2-3min before** session starts | **MOD** will warn for the session start. |
| We wait **around 2min** to let attendees join and start recording. | Get ready to take over **MOD** screen sharing once **CM** introduces you. |
| 2) Oral presentation phase | **CM** introduces you. Take over **MOD** screen sharing and get ready to talk once **CM** tells you to. Start your timer if one. |
|  | At **1min** (parallel sessions) or **5min** (plenary) of the end, if you are still far from the end of your presentation, **CM** will display a time notification on the shared screen. |
| End of the talk | **CM** will speak and start the question phase.  IMPORTANT: do not close your PPT/PDF and do not stop screen sharing until **MOD** takes over. |

| 3) Question phase | **CM** names the next **ATq**, and asks **ATq** to raise the hand if he/she would like to talk and CM reads the question meanwhile:  "**CM**: *We have a question from M. AttendeeQ; if you would like to talk, use the raise-the-hand button. The question was: How would you why...?*"  Wait a few seconds to see if **ATq** would like to add something and start answering. |
| --- | --- |
|  | **CM** checks if there is still some time left and eventually continues the question phase. |
| 4) End of presentation phase | Do not stop screen sharing or close your PPT/PDF until **MOD** takes over it.  Disable your microphone. |

## Troubleshooting

In case of issues under Zoom, see with your associated MOD (orally or by private chat).

In case of issue preventing you from using Zoom correctly, contact one of the following (in preference order):

**Relais Webcasting** contact:

Mathieu Lahargou

Relais Webcasting

Email: [*confidential*]

Phone or WhatsApp: [*confidential*]

**JOBIM** support contact:

Valentin Guignon

Email: [*confidential*]

Phone or WhatsApp: [*confidential*]

Skype: [*confidential*]

Teams: [*confidential*]
