# Supplementary material for: Ten simple rules for switching from face-to-face to remote conference: An opportunity to estimate the reduction in GHG emissions
Source: PLoS Comput Biol. 2021 Oct 18;17(10):e1009321. doi: 10.1371/journal.pcbi.1009321 (PMC8523038; doi:10.1371/journal.pcbi.1009321)
Supplement: S2 Table — (DOCX) [file pcbi.1009321.s004.docx]

## **S2 Table**: GHG emissions per session.

|  | **Duration**  **(min)** | **Number of attendees** | **Emissions (kg CO2e)** | | |
| --- | --- | --- | --- | --- | --- |
|  |  |  | **Network** | **Data center** | **Terminals** |
| **Opening** | 60 | 551 | 0.37 | 0.06 | 6.25 |
| **Keynote: Flora Jay** | 60 | 551 | 0.37 | 0.06 | 6.25 |
| **Phylogeny and Evolution** | 60 | 299 | 0.20 | 0.03 | 3.39 |
| **Networks and Systems** | 60 | 265 | 0.18 | 0.03 | 3.00 |
| **Keynote: Kjell Petersen** | 60 | 359 | 0.24 | 0.04 | 4.07 |
| **Platforms session** | 100 | 291 | 0.33 | 0.05 | 5.50 |
| **Statistics and Learning** | 80 | 307 | 0.28 | 0.05 | 4.64 |
| **Keynote: Martin Weigt** | 60 | 490 | 0.33 | 0.06 | 5.56 |
| **Mini symposium Long reads** | 150 | 490 | 0.83 | 0.14 | 13.89 |
| **Mini symposium Deep learning** | 150 | 289 | 0.49 | 0.08 | 8.19 |
| **Mini symposium RNA structure** | 150 | 83 | 0.14 | 0.02 | 2.35 |
| **Keynote: Johannes Söding** | 60 | 390 | 0.26 | 0.04 | 4.42 |
| **Functional genomics** | 60 | 173 | 0.11 | 0.02 | 1.96 |
| **NGS and Algorithmics** | 100 | 343 | 0.39 | 0.06 | 6.48 |
| **Keynote: Andrea Rau** | 60 | 412 | 0.28 | 0.05 | 4.67 |
| **SFBI general assembly** | 140 | 284 | 0.45 | 0.07 | 7.51 |
| **Computational Medicine** | 80 | 290 | 0.26 | 0.04 | 4.38 |
| **Proteins and Structure** | 120 | 239 | 0.32 | 0.05 | 5.42 |
| **Keynote: Nuria Lopez-Bigas** | 60 | 367 | 0.25 | 0.04 | 4.16 |
| **Closing** | 15 | 367 | 0.06 | 0.01 | 1.04 |
| **Total** |  | | **6.17** | **1.01** | **103.16** |
